# Tau Isoform-Regulated Schwann Cell Proliferation and Migration Improve Peripheral Nerve Regeneration After Injury

**DOI:** 10.3390/ijms252212352

**Published:** 2024-11-18

**Authors:** Shiying Li, Fuqian Zhang, Guifang Wang, Qianyan Liu, Xinghui Wang, Qianqian Chen, Dandan Chu

**Affiliations:** 1 Key Laboratory of Neuroregeneration of Jiangsu and Ministry of Education, Co-Innovation Center of Neuroregeneration, Nantong University, Nantong 226001, China; lisy0379@ntu.edu.cn (S.L.); zfq2024@sina.com (F.Z.); wangguifang1124@163.com (G.W.); lqyfamily@gmail.com (Q.L.); xhwang@ntu.edu.cn (X.W.); 2 NMPA Key Laboratory for Research and Evaluation of Tissue Engineering Technology Products, Nantong University, Nantong 226001, China; 3 Jiangsu Key Laboratory of Neuropsychiatric Diseases and Institute of Neuroscience, Soochow University, Suzhou 215123, China

**Keywords:** tau, alternative splicing, exon 4A, exon 10, Schwann cell, migration, peripheral nerve regeneration

## Abstract

Tau is a microtubule-associated protein that plays a vital role in the mammalian nervous system. Alternative splicing of the *MAPT* gene leads to the formation of tau isoforms with varying N-terminal inserts and microtubule-binding repeats. Dysregulation of tau alternative splicing has been linked to diseases in the central nervous system, but the roles of tau isoforms in the peripheral nervous system remain unclear. Here, we investigated the alternative splicing of tau exons 4A and 10 in the sciatic nerve and Schwann cells during development and following injury. We discovered that low-molecular-weight (LMW) tau, resulting from the exclusion of exon 4A, and 3R tau, generated by the exclusion of exon 10, diminishes with aging in rat sciatic nerve and Schwann cells. High-molecular-weight (HMW) tau and 3R tau increase in the adult sciatic nerve post-injury. We constructed viruses that expressed HMW−4R, LMW−4R, HMW−3R, and LMW−3R and introduced them into cultured cells or the distal part of the injured sciatic nerve to assess their effects on Schwann cell migration and proliferation. We also examined the effects of the four isoforms on axon growth and debris clearance after sciatic nerve injury. Our results demonstrated that tau isoforms inhibit Schwann cell proliferation while promoting Schwann cell migration and sciatic nerve regeneration. Specifically, the 3R−tau isoforms were more effective than the 4R−tau isoforms in promoting nerve regeneration. In conclusion, our study reveals the roles of tau isoforms in the peripheral nervous system and provides insights into the development of new therapeutic strategies for peripheral nerve injuries.

## 1. Introduction

Tau is one of the main microtubule-associated proteins found in the nervous system of mammals [1]. Human tau protein is encoded by the microtubule-associated protein tau (*MAPT*) gene on chromosome 17q21.31, which comprises 16 exons. In the central nervous system (CNS), tau isoforms with zero (0 N), one (1 N), or two (2 N) N-terminal inserts and three (3R tau) or four (4R tau) microtubule-binding repeats are created through alternative splicing of exons 2, 3, and 10 [2]. The longest isoform of human brain tau is 2N4R tau, with an apparent molecular weight of approximately 46 kDa [3]. In the peripheral nervous system (PNS), alternative splicing of tau exon 4A produces high-molecular-weight (HMW) tau isoforms with an apparent molecular weight of 110 kDa, also known as big tau [3,4]. The alternative splicing of tau is tightly regulated during development, and its dysregulation in the CNS is associated with the pathogenesis of neurodevelopmental and neurodegeneration diseases [5,6], while its roles in the PNS are yet to be studied.

Schwann cells are unique glial cells that play a vital role in the axon regeneration of the PNS. After peripheral nerve injury, the distal nerve stumps undergo Wallerian degeneration, which is characterized by the removal of axons and phagocytosis of myelin debris [7], while the proximal axon forms a growth cone and begins to regrow towards the distal end. At the same time, Schwann cells are recruited to the crushed area and undergo rapid reactive dedifferentiation, rapid proliferation, and migration to form the Büngner bands that provide axonal guidance. They also participate in the clearance of collapsed axons and myelin fragments, thus aiding in axon regeneration [8]. Therefore, the functions of Schwann cells are crucial for peripheral nerve regeneration and repair [9].

We have previously reported that tau is expressed in rat Schwann cells dissociated from the sciatic nerve. Gene silencing of *Mapt* was observed to increase Schwann cell proliferation, inhibit migration, and damage the regeneration of the peripheral nerve [10]. In this present study, we focused on investigating the alternative splicing of tau exons 4A and 10 in the sciatic nerve and Schwann cells during development and after injury. Further, we also analyzed the effects of tau isoforms on Schwann cell proliferation, migration, and nerve regeneration. The results revealed that tau promotes Schwann cell migration and sciatic nerve regeneration. In addition, the 3R−tau isoforms were found to be more effective than the 4R−tau isoforms.

## 2. Results

### 2.1. Tau Regulates the Repair of Rat Sciatic Nerve Injury

**Figure 1:**
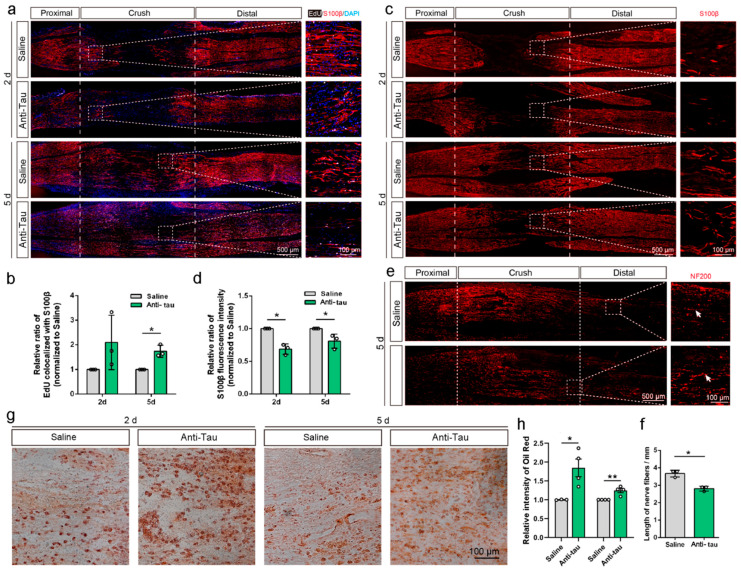
Tau antibody injection promoted Schwann cell proliferation and inhibited Schwann cell migration, axon growth, and myelin and lipid debris clearance following sciatic nerve injury. The rat sciatic nerve was injected with the same volume of saline or anti-tau antibody after being crushed. On the second (2 d) and fifth (5 d) day after the injury, the sciatic nerves were isolated and subjected to EdU (**a**), S100β (**c**), NF200 (**e**), or Oil Red O (**g**) staining. EdU, newly synthesized DNA. S100β, Schwann cells. DAPI, the nucleus. NF200, neurochemical marker. The box in the crush area is enlarged and displayed on the right panels. The relative intensity of EdU co-localized with S100β (**b**) and S100β (**d**) in the crush area (between two dashed lines in panel (**a**,**c**)) was calculated and analyzed (*n* = 3). (**e**,**f**) Sciatic nerves were immunostained with NF200 to observe axon elongation. The boxes of representative axons were enlarged and displayed on the right panels. The longest distance of grown axons was calculated and analyzed (*n* = 3). (**g**,**h**) Sciatic nerves were stained with Oil Red O (**g**) and the relative area was calculated in (**h**) (*n* = 3–4). The results were analyzed by Student’s *t*-test. *, *p* < 0.05; **, *p* < 0.01.

To investigate whether tau is involved in the regulation of sciatic nerve regeneration, we performed a crush injury on the sciatic nerve of adult rats and then injected Tau antibodies into the crushed area of the nerve. On the second and fifth days after injury, the tissue was dissected and subjected to immunofluorescence. Immunostaining for tau showed that the injection of the antibody significantly reduced tau levels in the crushed nerve (Supplementary Figure S1). An EdU (5-ethynyl-2′-deoxyuridine) assay revealed a significant increase in Schwann cell proliferation 5 days after injury in tau antibody-treated nerves (Figure 1a,b). However, Schwann cell marker S100β (Figure 1c,d) and neurochemical marker Neurofilament 200 (NF200) (Figure 1e,f) staining significantly decreased after tau antibody injection, indicating that the Schwann cell migration and axon growth was inhibited by tau down-regulation. The Oil Red O staining conducted to determine myelin and lipid debris clearance showed that more debris was accumulated in distal nerve stumps of tau antibody-injected rats (Figure 1g,h). These results suggested that the down-regulation of tau inhibited the recovery of rat sciatic nerve injury, which is consistent with our previous findings in tau knockout mice [10].

### 2.2. The Alternative Splicing of Tau Exons 4A and 10 is Differently Regulated in Newborn and Adult Schwann Cells Isolated from Rat Sciatic Nerve

**Figure 2:**
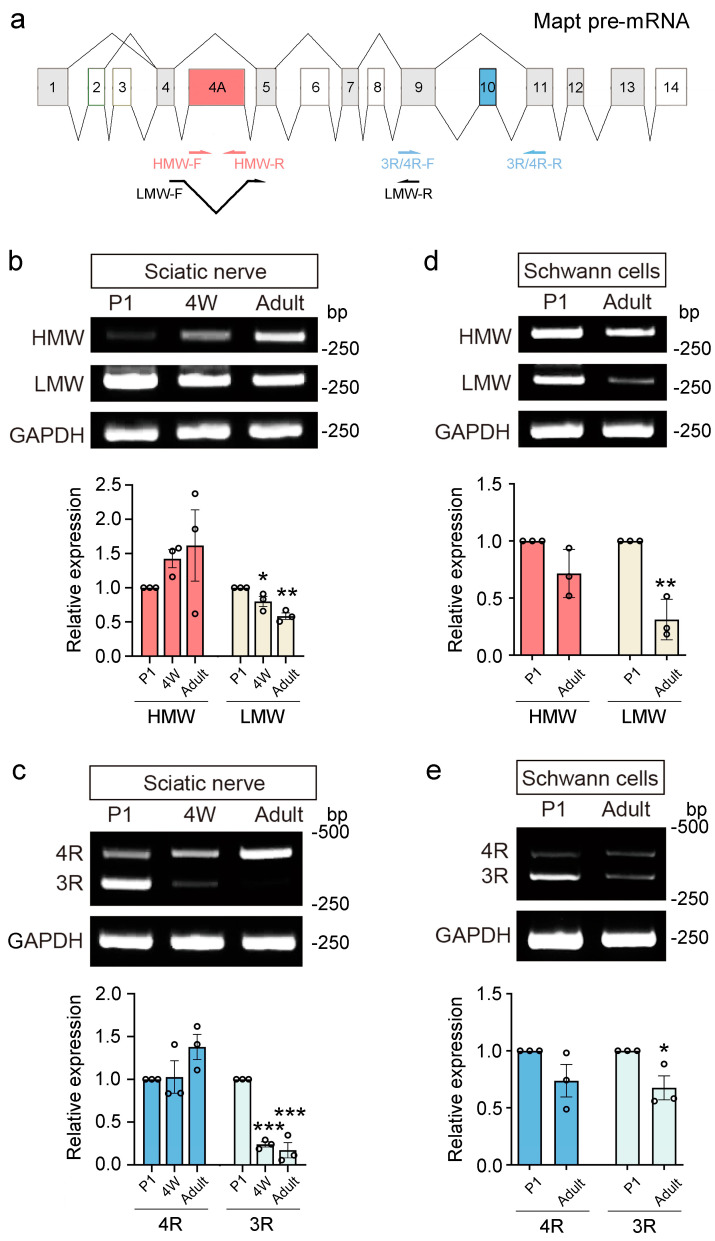
Alternative splicing of tau exons 4A and 10 in rat sciatic nerves and Schwann cells. (**a**) Schematics showing the alternative splicing of *Mapt* premRNA. HMW−F and HMW−R, LMW−F and LMW−R, and 3R/4R−F and 3R/4R−R indicated the location of the forward and reverse primers of HMW tau, LMW tau, and 3R/4R tau. (**b**) HMW tau, LMW tau, and (**c**) 3R/4R tau in the sciatic nerve stump of postnatal day 1 (P1), 4-week-old (4W), and adult rats were analyzed by RT-PCR. (**d**) HMW tau, LMW tau, and 3R/4R tau (**e**) in Schwann cells of P1 and adult rats. All data are presented as mean ± standard error (SEM) (*n* = 3). (**b**,**c**) One-way analysis of variance (ANOVA) followed by Dunnett’s multiple comparisons test. (**d**,**e**) Student’s *t*-test. *, *p* < 0.05. **, *p* < 0.01. ***, *p* < 0.001.

To further investigate the function of tau isoforms in the sciatic nerve, we first investigated the alternative splicing of tau exons 4A and 10 in the peripheral nerve by isolating mRNA from rat sciatic nerve stump and designing three pairs of primers, HMW tau, LMW tau (low-molecular-weight tau), and 3R/4R tau to detect tau transcripts including exon 4A, excluding exon 4A, and excluding or including exon 10, respectively (Figure 2a). RT-PCR analysis revealed that there was no significant difference in the expression of HMW tau in the sciatic nerve of the newborn (postnatal day 1, P1), 4-week-old, and adult rats (Figure 2b). However, the levels of LMW tau decreased to 80.0 ± 7.4% and 58.5 ± 4.8% in 4-week-old and adult rats (Figure 2b). We examined the transcription of tau exon 10 next and found that the levels of 4R tau in the sciatic nerve are not grossly changed during development, while the levels of 3R tau reduced to 24.0 ± 3.0% and 17.2 ± 8.9% (Figure 2c). These results show that the exclusion of tau exons 4A and 10 is down-regulated with age in rat sciatic nerves.

As one of the major cell types of the sciatic nerve stump [11], Schwann cells are reported to express tau [12]. Here, we isolated Schwann cells from P1 and adult rat sciatic nerves and analyzed the transcription of HMW tau, LMW tau, and 3R/4R tau. Similar to what was found in the sciatic nerve stump, there was no significant difference in the levels of HMW tau and 4R tau between P1 and adult in Schwann cells (Figure 2d,e). Meanwhile, the levels of LMW tau and 3R tau in adults decreased to 31.1 ± 10.3% and 68.3 ± 12.4%, respectively, compared to P1 rats (Figure 2d,e). These findings suggest that the exclusion of tau exons 4A and 10 is inhibited in adult Schwann cells, which is consistent with that in sciatic nerves.

### 2.3. The Alternative Splicing of Tau Exons 4A and 10 Is Changed Following Sciatic Nerve Injury

**Figure 3:**
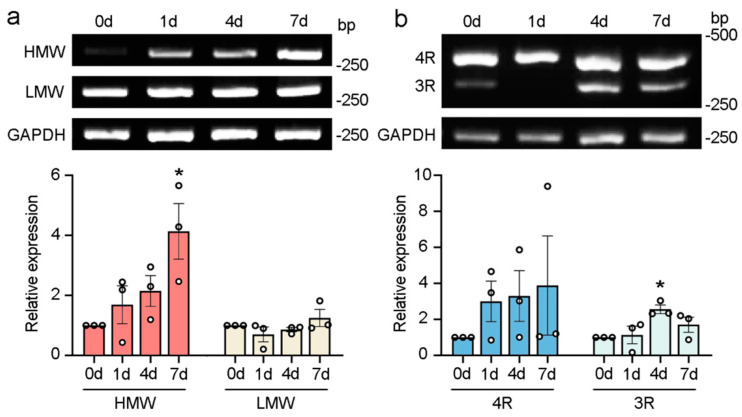
Sciatic nerve injury changes the alternative splicing of tau exons 4A and 10. mRNA of the sciatic nerve was isolated and analyzed by RT−PCR to detect the expression of HMW tau, LMW tau (**a**), and 3R/4R tau (**b**) 1, 4, and 7 days after a crush injury. Here, 0 days were used as uninjured controls. All data are presented as mean ± SEM (*n* = 3) and were analyzed by one−way ANOVA followed by Dunnett’s multiple comparisons tests, except for 4R tau. The expression of 4R tau was analyzed using the Kruskal–Wallis test. *, *p* < 0.05.

To examine whether the alternative splicing of tau exon 4A or 10 in peripheral nerve stumps was altered upon injury, we inflicted a crush injury on the sciatic nerves of the rats. Tau splicing in the sciatic nerves was detected at 1, 4, and 7 days post-injury. It was observed that HMW tau significantly increased 7 days after injury, while LMW tau showed little change from 0 days to 7 days (Figure 3a). The changes in 4R tau were not significant, while the levels of 3R tau were significantly elevated 4 days after injury (Figure 3b). These findings suggested that the alternative splicing of tau exons 4A and 10 is regulated following sciatic nerve injury.

### 2.4. The Effect of Tau Isoforms on the Proliferation and Migration of Cultured Schwann Cells

**Figure 4:**
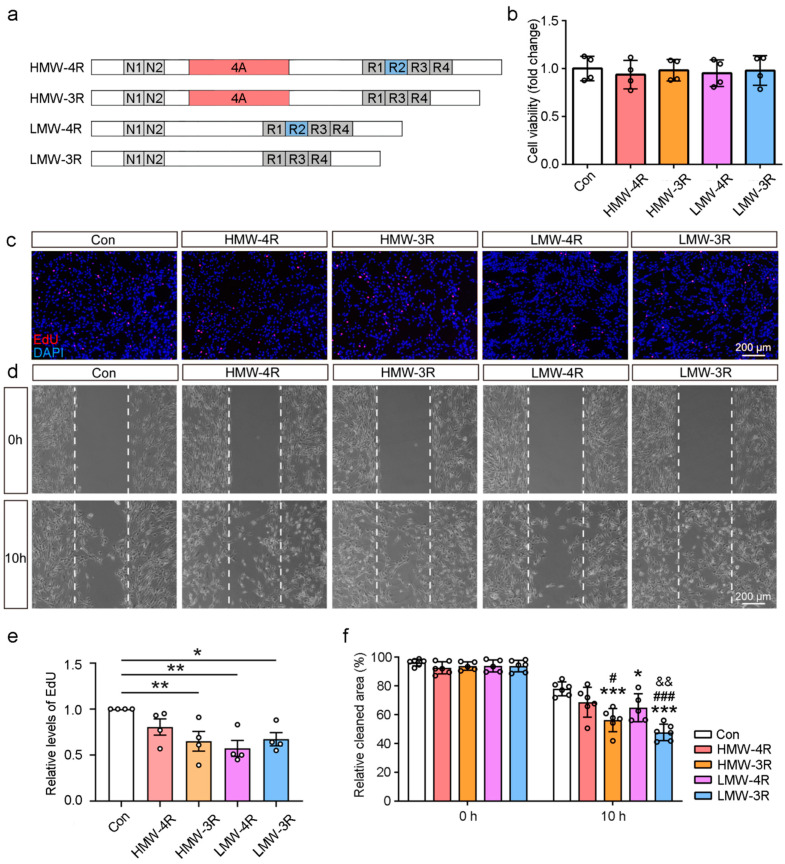
The effects of tau isoforms in inhibiting the proliferation and promoting migration of cultured Schwann cells. (**a**) Schematics of tau isoforms HMW−4R, HMW−3R, LMW−4R, and LMW−3R. N1 and N2, N-terminal inserts 1 and 2. 4A, tau exon 4A. R1~R4, the microtubule-binding repeats 1–4. (**b**) Schwann cells were infected with lentivirus expressing the four tau isoforms and examined for cell viability using the Cell Counting Kit-8 assay (*n* = 4). (**c**) Cell proliferation of the infected Schwann cells (*n* = 4) was determined by EdU and analyzed using the Kruskal–Wallis test followed by uncorrected Dunnett’s multiple comparisons test (**e**). DAPI (4′,6-Diamidino-2-Phenylindole) labeled the nucleus. (**d**) Schwann cells expressing the four isoforms were subjected to a wound healing assay. Ten hours after scratching, the clean area in the gaps was measured and analyzed (**f**). Con, *n* = 6. HMW−4R, *n* = 6. HMW−3R, *n* = 6. LMW−4R, *n* = 5. LMW−3R, *n* = 6. The results were analyzed by a one-way ANOVA followed by uncorrected Fisher’s LSD multiple comparisons test. *, compared to the control (Con). #, compared to HMW−4R. &, compared to LMW−4R. *, #, *p* < 0.05. **, &&, *p* < 0.01. ***, ###, *p* < 0.001.

Schwann cells are one of the primary cell types in peripheral nerve stumps where tau is expressed [12]. To better understand the effects of tau exons 4A and 10 on Schwann cell function, four types of lentiviruses expressing tau isoforms HMW−4R, HMW−3R, LMW−4R, and LMW−3R were constructed and used to infect primary Schwann cell cultures (Figure 4a). The results showed that lentivirus infection significantly enhanced the transcription of the corresponding tau isoforms (Supplementary Figure S2) and did not affect Schwann cell viability, as shown by the Cell Counting Kit-8 assay (Figure 4b). EdU (5-ethynyl-2′-deoxyuridine) staining indicated that HMW−3R, LMW−4R, and LMW−3R inhibited Schwann cell proliferation, but HMW−4R did not. However, no significant difference was found between the effects of HMW tau and LMW tau or 4R tau and 3R tau (Figure 4c,e).

Then, we used the wound healing assay to evaluate the roles of the four tau isoforms in Schwann cell migration and found that HMW−3R, LMW−4R, and LMW−3R, but not HMW−4R, significantly enhanced wound healing compared to the control (Figure 4d,f). Further, a two-way ANOVA analysis of the data of the four isoforms revealed that 3R−tau isoforms were more likely to promote Schwann cell migration than 4R−tau isoforms.

### 2.5. The Effects of Tau Isoforms on Schwann Cell Proliferation and Migration In Vivo

**Figure 5:**
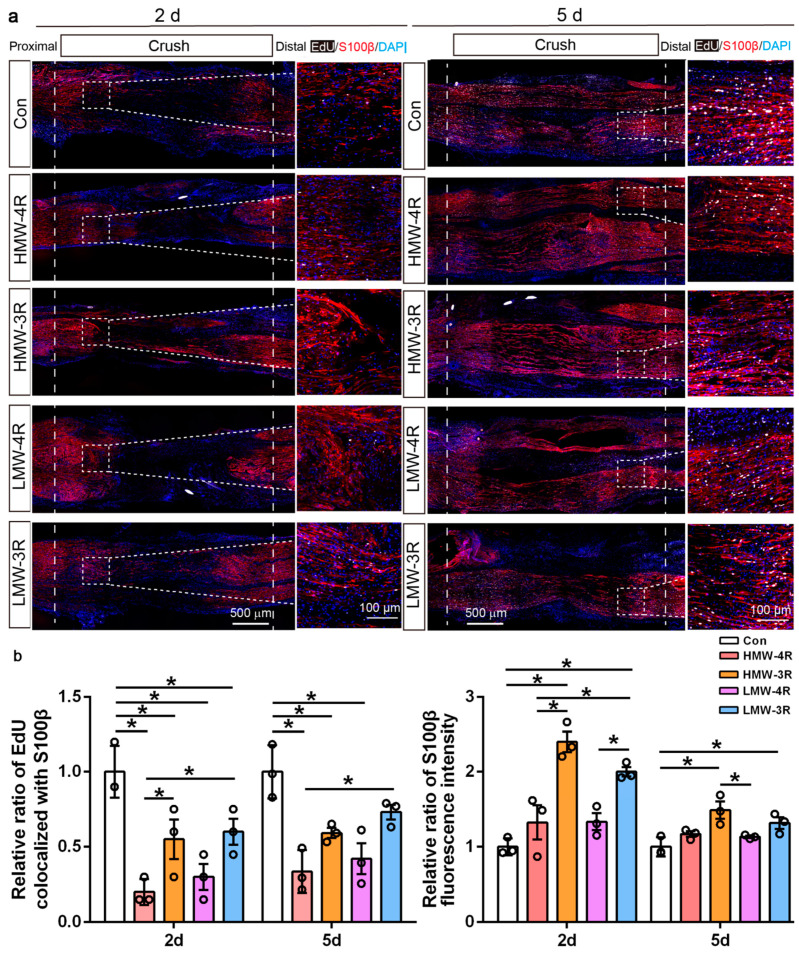
The effects of tau isoforms in regulating Schwann cell proliferation and migration following sciatic nerve injury. (**a**) Two weeks prior to the crush injury, lentiviruses expressing HMW−4R, HMW−3R, LMW−4R, or LMW−3R were injected into the sciatic nerve in advance of undergoing a crush injury. On the second (2 d) and fifth (5 d) day after the injury, the sciatic nerves were isolated and subjected to EdU and S100β staining. EdU, newly synthesized DNA. S100β, Schwann cells. DAPI, the nucleus. The box in the crush area is enlarged and displayed on the right panels. (**b**) The relative intensity of S100β and EdU co-localized with S100β in the crush area (between two dashed lines in (**a**) were calculated and analyzed (*n* = 3). The results were analyzed by a one-way ANOVA followed by Tukey’s multiple comparisons test. *, *p* < 0.05.

To elucidate the roles of tau exons 4A and 10 in regulating cell proliferation and migration in vivo, lentiviruses expressing the four tau isoforms were injected into the crushed area of the sciatic nerve, and a crush injury was inflicted on the sciatic nerves 2 weeks after virus injection. The expression levels of tau isoforms were comparable among the four groups (Supplementary Figure S3). Schwann cell proliferation and migration were examined 2 days and 5 days after injury (Figure 5a). Calculation of EdU signals co-localized with Schwann cell marker S100β showed that on the second day after the injury, all four isoforms inhibited Schwann cell proliferation significantly; on the fifth day after the injury, HMW−4R, HMW−3R, and LMW−4R were able to reduce proliferation (Figure 5b). A two-way ANOVA analysis suggested that either on day 2 or day 5, 4R−tau isoforms were more prone to inhibit Schwann cell proliferation than 3R−tau isoforms.

On both the second and fifth day after the injury, expression of HMW−3R and LMW−3R significantly increased the number of S100β-positive cells in the crush area without alternation in Schwann cell proliferation, indicating a promoted migration of Schwann cells to the injury site (Figure 5a,b). A two-way ANOVA analysis suggested that 3R−tau isoforms but not 4R−tau isoforms elevated Schwann cell migration after injury.

### 2.6. Tau Isoforms Improve Sciatic Nerve Regeneration After Injury

**Figure 6:**
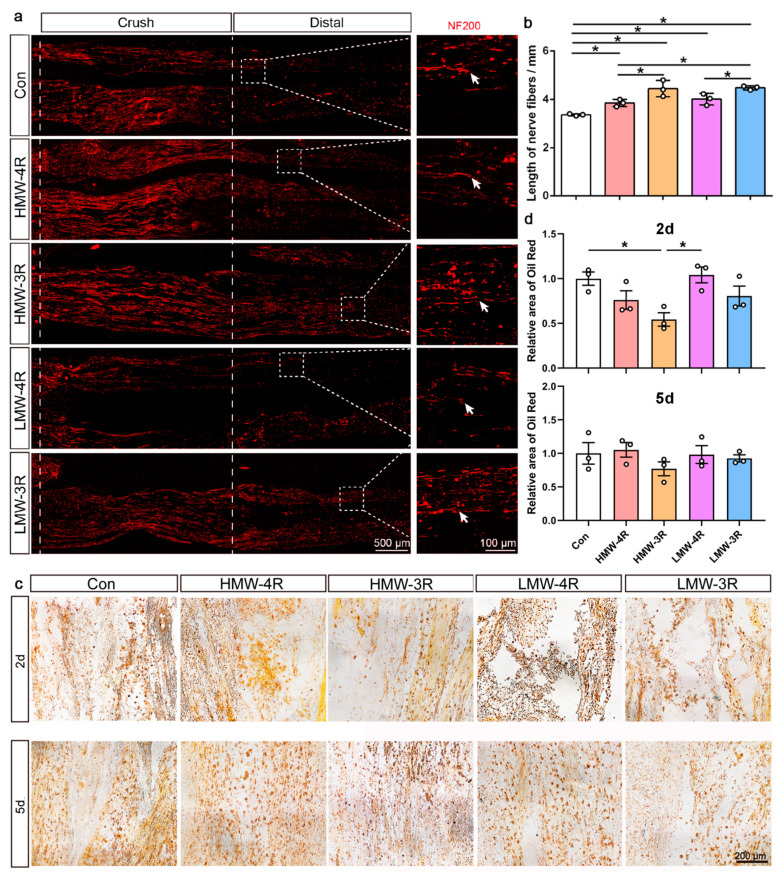
Tau isoforms improve sciatic nerve regeneration. (**a**) The distal end of the rat sciatic nerve was injected with lentiviruses expressing HMW−4R, HMW−3R, LMW−4R, or LMW−3R. Two weeks after the injection, the rats underwent sciatic nerve injury. On the fifth day after the injury, the sciatic nerves were isolated and subjected to NF200 staining. The box in the distal region is enlarged and displayed on the right panels. An arrow is used to mark the growing end of the axon fibers. (**b**) The growth distance of NF200 in the distal area was calculated and analyzed (*n* = 3). The results were analyzed by a one-way ANOVA followed by Tukey’s multiple comparisons test. (**c**) Oil Red O staining showed the clearance of debris in the distal region of the sciatic nerve at 2 days or 5 days after injury. (**d**) The relative intensity of Oil Red in panel (**c**) was qualified and analyzed by a one-way ANOVA, followed by Tukey’s multiple comparisons test (*n* = 3). *, *p* < 0.05.

Following the injury, we proceeded with immunofluorescence staining on the sciatic nerve after 5 days and utilized NF200 to label the axons in order to assess their regeneration. Our findings indicated that all four tau isoforms enhanced the length of the sciatic nerve fiber in the digital region, with 3R−tau isoforms demonstrating superior results (Figure 6a,b). Furthermore, Oil Red O staining revealed that HMW−3R-injected rats had less debris accumulated in the distal nerve stumps 2 days after injury (Figure 6c,d). However, no significant differences in fragment clearance between the groups were observed at 5 days post-injury (Figure 6c,d). These results suggest that tau isoforms, especially 3R−tau isoforms, have a positive impact on the regeneration of the sciatic nerve after an injury.

## 3. Discussion

As one of the major microtubule-associated proteins in the mammalian nervous system, the primary function of tau is to stabilize microtubules and promote their assembly [13]. However, it has been found that retinal neuron survival and axonal regeneration after injury are not influenced by tau gene deletion [14]. In the present study, tau antibodies are used to reduce tau levels in the sciatic nerve. In contrast to the findings in the CNS, our results show increased proliferation and decreased migration of Schwann cells, as well as slower repair of the axon. This is consistent with previous results in *Mapt* siRNA-treated rats and *Mapt* knockout mice [10]. On the contrary, overexpression of specific tau isoforms facilitates the repair of peripheral nerve injury. These findings suggest that tau plays an essential role in injury repair of peripheral nerves through the regulation of Schwann cells.

HMW tau (also known as big tau) generated by the inclusion of exon 4A was discovered to be expressed in adult PNS neurons such as dorsal root ganglion (DRG) neurons [15] and certain populations of CNS neurons [3]. It is believed that HMW tau can improve axonal transport and potentially prevent tau aggregation in specific pathologic conditions [16]. In the dorsal root, LMW tau is present throughout all DRG neurons in embryonic and neonatal rats. However, HMW tau is not initially expressed in DRG neurons until it reaches the adult level at P14 [4], unlike in Schwann cells where it is present throughout the neonatal stage and adulthood. The transcription of HMW tau in the sciatic nerve from P1 to adult stages also showed no significant changes, similar to that found in Schwann cells. Additionally, it has been reported that HMW tau decreases in DRG after sciatic nerve injury, with the most significant reduction occurring at P7 [17]. In contrast to DRG neurons, the levels of HMW tau at the injured site increase rather than decrease after sciatic nerve injury in the present study, indicating that the function of HMW tau in Schwann cells may be distinct from that in neurons.

The alternative splicing of tau exon 10 has been extensively researched in the CNS, but it is not as well studied in the PNS [18]. In the human brain, 3R−tau isoforms are expressed throughout life, while 4R−tau isoforms are specifically expressed in adults [2]. However, in rodent brains, 3R tau is highly expressed only in fetuses and infancy, while 4R tau is mainly expressed in adulthood [5,19]. Here, we elucidate in the rat sciatic nerve and Schwann cells that 3R gradually decreases from neonatal to adulthood. Unlike in CNS, the changes in 4R tau in the sciatic nerve and Schwann cell are not significant between infancy and adulthood. Regarding the changes in tau exon 10 splicing after sciatic nerve injury, one group reported that both the 3R− and 4R−tau mRNA on the spinal cord decreased and then returned to the contralateral levels by 7 days after being crushed [20]. However, we find that, differently from in the spinal cord, the levels of 3R tau are significantly increased by day 4 after the crush in the sciatic nerve. Also, overexpression of 3R tau shows a better repair of the nerve injury than 4R tau. Based on these results, we conclude that tau exon 10 splicing in the PNS glia cells–Schwann cells is different from that in CNS or in neurons during development and nerve injury. Exclusion of tau exon 10 in Schwann cells could benefit the regeneration of the peripheral nerve.

The transfection of LMW−4R tau has been shown to activate cultured rat microglia and promote migration and phagocytosis, potentially by synergistically regulating the dynamics of the actin cytoskeleton [21]. We have constructed four tau isoforms with or without exon 4A or exon 10, among which LMW−4R is sufficient to promote cell migration in cultured Schwann cells. However, both HMW−3R and LMW−3R significantly stimulate the migration of Schwann cells both in vitro and in vivo. We speculate that the weaker effect of 3R tau on microtubule stabilization compared to 4R tau [22,23] may promote cell migration through the synthesis of more dynamic microtubules.

According to research conducted by Sennvik et al., the depletion of tau has been found to induce hippocampal neuron proliferation [24]. On the other hand, LMW−4R tau expression has been shown to suppress neuronal proliferation and promote differentiation in *Mapt* knockout mice [24], likely by directly interacting with phosphoinositide 3-kinase (PI3K) and impairing PI3K-AKT signaling that is necessary for neuron proliferation [25]. Similar findings have been observed here in Schwann cells, where tau isoforms, particularly 4R−tau isoforms, inhibit Schwann cell proliferation and synergistically facilitate sciatic nerve regeneration with 3R−tau-stimulated cell migration.

We found no significant differences in the regulation of Schwann cell proliferation and migration by HMW−tau and LMW−tau isoforms. Nevertheless, further investigation is required to reveal the specific roles of HMW tau. In conclusion, our results suggest that tau exon 10 plays a more crucial role in repairing peripheral nerve injuries compared to exon 4A. These findings underscore the significance of understanding tau splicing in the peripheral nervous system and its potential implications for treating peripheral nerve injuries.

## 4. Materials and Methods

### 4.1. Animals

Sprague Dawley rats were obtained from the Experimental Animal Center of Nantong University of China. The rats were randomly assigned to groups and housed in cages with two other rats. They had access to food and water ad libitum and followed a 12 h light and dark cycle. The study used neonatal 1-day-old rats (both male and female), 4-week-old males, and male adults weighing 200–220 g. All procedures conducted were ethically approved by the Administration Committee of Experimental Animals and followed the Institutional Animal Care Guidelines of Nantong University.

### 4.2. Sciatic Nerve Crush Surgery

The rats were anesthetized by intraperitoneal injections of 0.35 mL per 100 g body weight of a narcotics mixture (trichloroacetaldehyde monohydrate 85 mg/kg, magnesium sulfate 42 mg/kg, and sodium pentobarbital 17 mg/kg) (Ruijie, Shanghai, China). Then, they underwent a surgical procedure to crush the left sciatic nerve as previously described [26]. Briefly, the skin of the lateral aspect of the midthigh of the left hind limb was incised to expose the sciatic nerve. The 3 mm sciatic nerve was crushed three times with forceps (54 N), each lasting for 10 s. The uncrushed nerve served as the control. The sciatic nerve was harvested at certain intervals after the surgery for further analysis using RT-PCR or immunofluorescence.

### 4.3. Antibody and Virus Injection

Briefly, 5 μL of 0.9% saline (as a control) or 5 μL of anti-tau antibodies (ab64193, Abcam, Cambridge, UK) was mixed with an equal volume of Matrigel (Corning, Corning, NY, USA) and injected into the injured sites instantly after the crush injury to the left sciatic nerve of rats. The antibody specifically targets the N-terminal region of tau protein (5–12 a.a.), thus recognizing all isoforms of tau. At 2 days and 5 days post-surgery, rat sciatic nerves were harvested for further analysis by immunofluorescence.

Four types of lentiviruses were constructed by Genechem Co., LTD. (Shanghai, China), expressing tau isoforms HMW−4R, HMW−3R, LMW−4R, or LMW−3R, all fused with an enhanced green fluorescent protein (EGFP). Briefly, DNA fragments of different tau isoforms were synthesized and cloned into the vector Ubi-MCS-SV40-EGFP-IRES-puromycin. The sequences were validated through DNA sequencing. The expression vector and helper plasmids were co-transfected into human embryonic kidney HEK 293T cells. Forty-eight hours post-transfection, the virus was harvested from the supernatant, and its titer was quantified. The crushed area was injected with 1 × 10^6^ transducing units of lentivirus, and the sciatic nerves were subjected to a crush injury two weeks after the virus injection.

### 4.4. Reverse Transcription–PCR (RT-PCR)

We used RT-PCR to detect tau isoforms primarily because it allows for direct visualization and approximate measurement of band molecular weight for assessing primer specificity, while also enabling simultaneous amplification of the 3R−tau and 4R−tau bands in one single reaction. RNA was isolated from the sciatic nerve or cultured Schwann cells using Trizol Reagent (Life Technologies, Carlsbad, CA, USA) per the manufacturer’s instructions. The quality of the RNA samples was assessed with the Agilent Bioanalyzer 2100 (Agilent Technologies, Santa Clara, CA, USA), and the NanoDrop ND-1000 spectrophotometer (Infinigen Biotechnology Inc., City of Industry, CA, USA) was used to determine the quantity of RNA. The extracted RNA was reverse-transcribed to complementary DNA (cDNA) by the Omniscript Reverse Transcription Kit (Qiagen, Valencia, CA, USA). Taq DNA polymerase was purchased from Vazyme (P222). The PCR program was as follows: 95 °C for 5 min; 35 cycles of 95 °C for 40 s, 60 °C for 30 s, and 72 °C for 45 s; and a dissociation cycle consisting of 72 °C for 5 min. The sequences of primer pairs can be found in Table 1. The PCR products were resolved on 1–2% agarose gels and quantitated using the ImageJ software (1.48v, National Institutes of Health, Bethesda, MD, USA). We selected equally sized regions of interest (ROIs) for each band to ensure consistent measurement of their grayscale values during analysis.

### 4.5. Cell Culture

Schwann cells were isolated from the sciatic nerve stumps at specific time points and purified to remove fibroblasts as described [27]. The Schwann cells were cultured in Dulbecco’s Modified Eagle Medium (Invitrogen, Carlsbad, CA, USA) containing 10% fetal bovine serum (Invitrogen, Carlsbad, CA, USA), 1% penicillin and streptomycin (Invitrogen, Carlsbad, CA, USA), 2 μM forskolin (Sigma, St. Louis, MO, USA), and 10 ng/mL heregulin (Millipore Sigma, Burlington, MA, USA) in a humidified 5% CO_2_ incubator at 37 °C. For cell transfection, primary cultured Schwann cells were infected with an equal titer of lentiviruses expressing tau isoforms by using a Lipofectamine RNAiMAX transfection reagent (Invitrogen, Carlsbad, CA, USA) 48 h after transfection.

### 4.6. Cell Proliferation Assay

Primary Schwann cells were seeded at 2 × 10^5^ cells/mL density onto 96-well plates coated with 0.01% poly-L-lysine. After 36 h of infection, the cell culture medium was supplemented with 50 μM EdU, and the cells were allowed to grow for another 12 h. The cells were then fixed with 4% paraformaldehyde and measured for cell proliferation using the Cell-Light EdU DNA Cell Proliferation Kit (RibiBio, Guangzhou, China). The nucleus was labeled with Hoechst 33,342 (RibiBio, Guangzhou, China). Images were taken using a Leica DMR fluorescence microscope (Leica Microsystems, Bensheim, Germany). For the quantification of EdU levels, 4 independent repetitions were performed. In each experiment, at least 4 views were randomly selected from each group. The levels of EdU were calculated by dividing the number of EdU-positive nuclei by the total number of nuclei in the randomly selected images and then normalizing the results to the control group. The EdU levels from the independent views for each group were averaged to obtain the relative EdU levels for further statistical comparisons.

In order to identify cell proliferation in vivo, the rats were injected with a concentration of 5 mg/kg EdU (C10340, Invitrogen, Carlsbad, CA, USA) dissolved in saline intraperitoneally 24 h before dissection. The injured sciatic nerve was collected, fixed with 4% paraformaldehyde, and sliced at a thickness of 12 µm for further immunofluorescence staining.

### 4.7. Wound Healing Assay

The purified Schwann cells were cultured with a complete medium in the scratch molds (Ibidi, Martinsried, Germany), infected with lentiviruses expressing tau isoforms. The scratch mold was removed 48 h after infection. The cells were rinsed with phosphate-buffered saline to remove debris and incubated with a low-serum medium (2% fetal bovine serum) containing 0.15 μg/mL Mitomycin C. Images were taken at 0 h and 10 h after removing the mold using a DMR fluorescence microscope (Leica Microsystems, Bensheim, Germany). The relative cleaned area was determined by dividing the clean area by that of the control at 0 h from randomly selected images.

### 4.8. Immunofluorescence

The sciatic nerve or cultured cells were fixed with 4% paraformaldehyde, washed with phosphate-buffered saline 3 times, blocked with immunostaining blocking buffer (Beyotime, Shanghai, China), incubated with anti-S100β antibodies (1:100, ab52642, Abcam, Cambridge, UK) or anti-NF200 antibodies (1:200, N2912, Sigma-Aldrich, St. Louis, MO, USA), and then detected with Cy3-conjugated secondary antibody (SA00009-1, 1:400, Proteintech, Rosemont, IL, USA). The nuclei were labeled using DAPI Fluoromount-G (SA00009-1, 1:400, Proteintech, Rosemont, IL, USA). Images were captured using an Axio Imager M2 epifluorescence microscope (Carl Zeiss Microscopy, Jena, Germany). The fluorescence intensity in the 3 mm injured sciatic nerve was measured and compared.

### 4.9. Oil Red O Staining

Oil Red O staining was performed to determine the effect of tau isoforms on degenerated myelin debris clearance rates. Briefly, sciatic nerve sections were fixed in 10% formalin and then washed 3 times with a 0.01 M PBS solution for 5 min each. Subsequently, they were sequentially washed with 60% isopropanol. The sections were then incubated in 0.6% Oil Red O (Sigma, St. Louis, MO, USA) for 15 min at 37 °C. After the staining process, the slices were transferred to 0.01 M phosphate-buffered saline (PBS) and washed 3 times for 5 min each to diminish any background interference. Finally, the slices were labeled using glycerin, and images were taken under a Zeiss Axio Imager M2 microscope (Carl Zeiss Microscopy GmbH, Jena, Germany). ROIs of the same size were randomly selected from the damaged area, and the area occupied by Oil Red O staining was quantitated using Adobe Photoshop 11.0.2 (San Jose, CA, USA).

### 4.10. Statistical Analysis

All data are presented as mean ± SEM and were analyzed by GraphPad Prism 9.0.2 (GraphPad Software Inc., San Diego, CA, USA). The data were first analyzed using the Shapiro–Wilk test for their distribution. Student’s *t*-test was performed to analyze differences between two groups of normally distributed data (*p* > 0.05 in the Shapiro–Wilk test). A one-way ANOVA followed by Tukey’s post hoc test was used for multiple comparisons among normally distributed data (*p* > 0.05 in the Shapiro–Wilk test). The Kruskal–Wallis test was used for multiple data groups found not normally distributed (*p* < 0.05 in the Shapiro–Wilk test). Dunnett’s, Tukey’s, or uncorrected Fisher’s LSD multiple comparisons tests were used for post hoc tests as indicated in the figure legends. A two-way ANOVA was used to analyze the difference between HMW tau and LMW tau or 4R tau and 3R tau. *p* < 0.05 was considered statistically significant.

## Figures and Tables

**Table 1:** The sequences of primers used in this study.

Primer		Sequence
HMW tau	Forward	5′-TCTCTGGGGAGACCACTAGC-3′
HMW tau	Reverse	5′-GCAGGTTGCTTGTCAGTTGG-3′
LMW tau	Forward	5′-CTCAAGCTCGAGTGGCCG-3′
LMW tau	Reverse	5′-GTTGGTAGGGATGGGGTACG-3′
3R4R	Forward	5′-GGTGAACCACCAAAATCCGGAGAACG-3′
3R4R	Reverse	5′-CCACACTTGGAGGTCACCTTGC-3′
GAPDH	Forward	5′-ACAGCAACAGGGTGGTGGAC-3′
GAPDH	Reverse	5′-TTTGAGGGTGCAGCGAACTT-3′

## Data Availability

The data that support the findings of this study are available from the corresponding author upon reasonable request.

## Supplementary Materials

The following supporting information can be downloaded at https://www.mdpi.com/article/10.3390/ijms252212352/s1.
