# Supplementary material for: Tau Isoform-Regulated Schwann Cell Proliferation and Migration Improve Peripheral Nerve Regeneration After Injury
Source: Int J Mol Sci. 2024 Nov 18;25(22):12352. doi: 10.3390/ijms252212352 (PMC11594695; doi:10.3390/ijms252212352)
Supplement: Supplementary file 1 [file ijms-25-12352-s001.zip › ijms-3276144-supplementary.pdf]

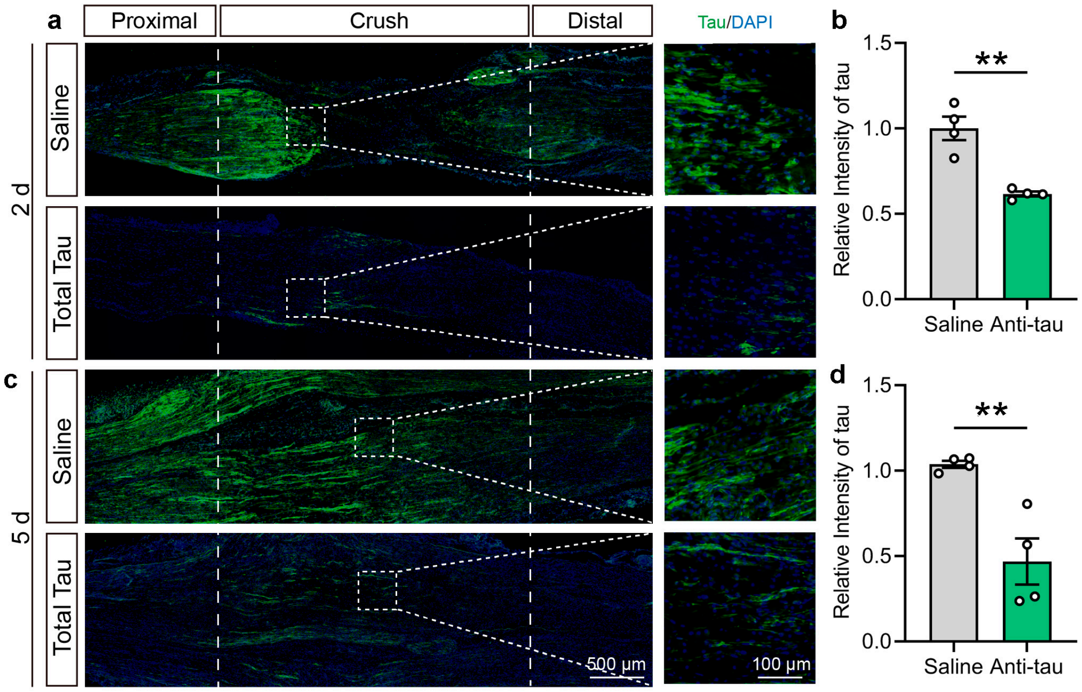

Supplementary Figure S1: The injection of tau antibody led to a reduction in tau levels in the crushed sciatic nerve. A crush injury was induced on the sciatic nerve of adult rats, followed by the injection of tau antibody into the damaged area (with saline as a control). Tissue samples were collected on the second (a-b) and fifth days (c-d) post-injury using immunofluorescence to detect tau. A region of interest (ROI) measuring 1000 x 1000 pixels in the proximal area of the crushed nerve was used to quantify tau intensity, as shown in panels (b) and (d). The data were presented as mean ± SEM (n = 4) and analyzed by Student's *t* test. **, $P < 0.01$.

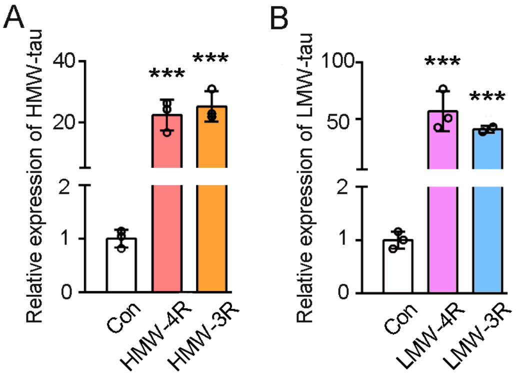

Supplementary Figure S2: The expression levels of tau isforms in Schwann cells. The cells were infected with lentivirus expressing HMW-4R, HMW-3R, LMW-4R, or LMW-3R. The mRNA levels of the four isoforms were analyzed by RT-PCR. All data were presented as mean ± SEM (n = 3) and analyzed by one-way ANOVA followed by Dunnett's multiple comparisons tests. ***, $P < 0.001$.

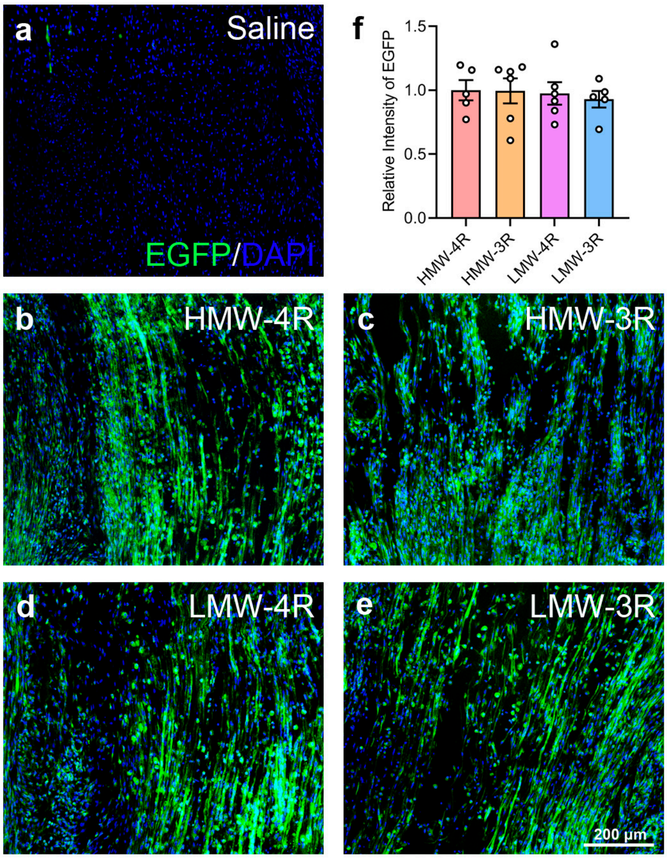

Supplementary Figure S3: The expression of tau in the sciatic nerve following lentivirus injection. Equal titers of lentivirus carrying the vector Ubi-MCS-SV40-EGFP-IRES-puromycin, which expressed different tau isoforms fused to EGFP, were injected into the sciatic nerve 2 weeks before a crush injury. Five days post-injury, the nerve tissue was harvested and the expression of tau was assessed by EGFP signal. The intensity of EGFP in the 3 mm crushed region was quantified and presented in panel (f). The data are expressed as mean ± SEM and were analyzed using one-way ANOVA. Sample sizes were as follows: HMW-4R (n = 5), HMW-3R (n = 6), LMW-4R (n = 6), and LMW-3R (n = 5).
